# Validity and Reliability of a New Optoelectronic System for Measuring Active Range of Motion of Upper Limb Joints in Asymptomatic and Symptomatic Subjects

**DOI:** 10.3390/jcm8111851

**Published:** 2019-11-02

**Authors:** Rodrigo Martín-San Agustín, Jose A. García-Vidal, German Cánovas-Ambit, Aurelio Arenas-Della Vecchia, Manuel López-Nicolás, Francesc Medina-Mirapeix

**Affiliations:** 1 Department of Physiotherapy, University of Valencia, 46010 Valencia, Spain; rodrigo.martin@uv.es; 2 Department of Physiotherapy, University of Murcia, Campus de Espinardo, 30100 Murcia, Spain; mirapeix@um.es; 3 Research group Fisioterapia y Discapacidad, Murcia BioHealth Research Institute-Virgen de la Arrixaca Hospital, El Palmar, 30120 Murcia, Spain; 4 Research fellow, Department of Physiotherapy, University of Murcia, Campus de Espinardo, 30100 Murcia, Spain; ambitcanovasgerman@gmail.com; 5 Department of Electromagnetism and Electronics, University of Murcia, Campus de Espinardo, 30100 Murcia, Spain; arenas@um.es; 6 Department of Dermatology, Stomatology, Radiology and Physical Medicine, University of Murcia, 30100 Murcia, Spain; mlopeznicolas@gmail.com

**Keywords:** range of motion, upper limb joints, biomedical technology, optoelectronic device

## Abstract

The aim of this study was to evaluate the validity of the Veloflex infrared dynamic angle-meter (Veloflex-IDA) and the intra- and inter-rater reliability when measuring the ranges of motion (ROMs) of the upper limb joints. Thirty-five healthy and 20 symptomatic participants were evaluated. Twelve upper limb movements were measured in two sessions with the Veloflex-IDA, which is a device composed of a camera that tracks the trajectory of retro-reflective markers. In addition, a goniometer was used in the first session to evaluate concurrent validity. Validity and agreement were evaluated by Pearson correlation coefficient (r) and Bland–Altmann plots. Intra- and inter-rater reliability were evaluated using intra-class correlation (ICC), standard error of measurement (SEM), and minimal detectable change (MDC). Both instruments showed excellent correlation for all movements (*r* range from 0.992 to 0.999). The intra- and inter-rater reliability were excellent (ICC range from 0.95 to 0.99 and 0.90 to 0.98, respectively). Intra-rater reliability showed SEMs <1.38% and <5.19% and inter-rater reliability SEMs <2.26% and <5.22% for asymptomatic and symptomatic, respectively. Veloflex-IDA is a valid and reliable alternative to measure the upper limb joints’ ROM and it can be used in clinical practice and research after basic training.

## 1. Introduction

The evaluation of the range of motion (ROM) is usually carried out by physiotherapists and physical trainers to quantify the joint movement and to obtain useful information to help them direct their interventions. Thus, altering the upper limb joint’s ROM can limit functionality in patients with musculoskeletal and neurological disorders [1,2,3] and increase the risk of injury in different sports [4,5].

The goniometer is the most used and standardized method for ROM measuring [6]. Even though it is a low cost and easy-to-use instrument, its application in the upper limb’s ROM analysis has been criticized because its need for the examiner to have a high level of experience [7]. For this reason, multiple tools have emerged as alternatives to quantify the ROM, some of which are easy to use, such as digital inclinometers [8,9], smartphone applications [10,11,12], laser-guided digital goniometers [13], or electrogoniometers [14] and others of more laborious to use such as capture software from images [15] or optoelectronic devices [16].

While the first optoelectronic devices are generally more expensive or need calibration time (15–20 min) [17], the development of new simpler optoelectronic systems such as Veloflex infrared dynamic angle-meter (Veloflex-IDA) has facilitated its use, since it does not need calibration or processing after the movement. Therefore, the aim of this study was to evaluate the validity of the Veloflex-IDA and the intra- and inter-rater reliability in its use to measure the ROMs of the upper limb joints.

## 2. Methods

### 2.1. Participants

Thirty healthy volunteers and 25 subjects with joint disorder in the upper limb were recruited at a sports center in Murcia, Spain. Inclusion criteria for the healthy group were having no history of pathology, surgery, or permanent impairments in the upper limb and having no pain in any of the joints examined. Symptomatic participants were included if they reported an average pain of ≥3/10 at rest and if they had limited mobility of some upper limb joint for more than 3 weeks. All those who initially contacted the researchers to participate in the study were ultimately examined in the same sports center.

Once the study procedures were explained to the participants in detail, they signed an informed consent and completed an information sheet prior to data collection (demographic and anthropometric data). The study was approved by the research ethics committee of the University of Murcia (CEI-2263).

### 2.2. Procedures

An experienced examiner in the measurement techniques evaluated the participants in two sessions, with a 1-week interval between sessions for the healthy group. He was a physiotherapist who had used goniometry methods for 10 years in clinical practice. In addition, a second examiner (newly graduated physiotherapist) without experience was instructed in the procedures and performed the same tests in the second session. Symptomatic participants were analyzed by both examiners with the Veloflex-IDA on the same day (with a 1-h interval between measurements) to minimize the influence of their changes in symptoms on the reliability of the device. The order in which the examiners measured was randomized and an assistant researcher read Veloflex-IDA values at all sessions in order to avoid bias. All measurements were performed in the same laboratory and time of the day and in a similar temperature environment in both sessions.

**Figure 1:**
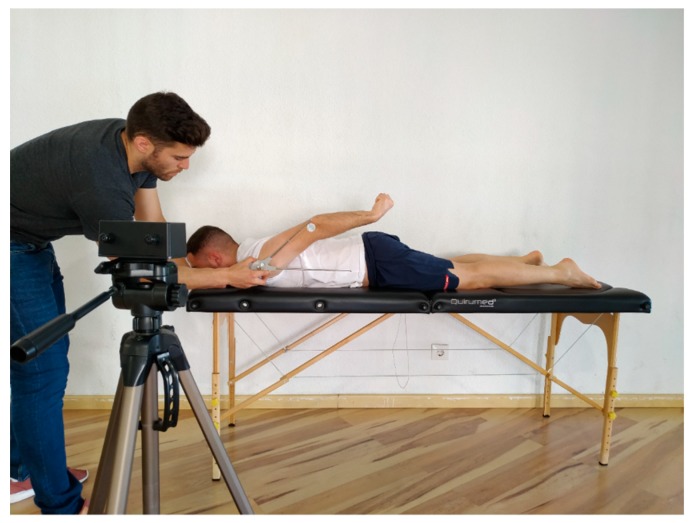
Measurement of shoulder joint range of motion for extension.

In the first session, all movements were measured by a simple long-arm goniometer with a 360° scale marked in one-degree increments (Orthopedic Equipment Co., Bourbon, IN, USA) and Veloflex-IDA (Veloflex-IDA developed by Deportec, Murcia, Spain) in order to examine concurrent validity. In the second session, only the Veloflex-IDA was used by both examiners to examine intra and inter-rater reliability. Veloflex-IDA is an optoelectronic system composed of one infrared cam with a tripod, a laptop, and markers easily placed on the skin. The camera allows to register the markers’ movement trajectories in each moment and, in this way, to register the ROM throughout its trajectory. To do this, the camera is placed on an adjustable tripod placed 1–1.5 m away from the participant and located at the height of the examined joint (Figure 1). Veloflex-IDA can determine the angle formed by two bone segments (taking three reference points: one joint axis and two references), or alternatively between a bone segment and the horizontal/vertical line drawn on the joint axis. Depending on the proximity of the camera to the joint, the system’s accuracy was reported between 0.1° and 1°. This point tracking by a camera system facilitates automatic ROM detection, minimizing time and steps compared to other optoelectronic devices. It has also the advantage over traditional tools (e.g., goniometer) or mobile phones to free the evaluator from having to stand next to the patient.

In each session, 12 movements were examined. The testing order was shoulder, elbow, and wrist movements. For the shoulder, the order was randomized, but pairs of movements on the same plane (e.g., flexion and extension, internal, and external rotation etc.) were always measured consecutively. All test positions and references for goniometer alignments were chosen according to previous authors’ recommendations [18]. These references were also used for Veloflex-IDA’s markers. Prior to the measurement of each movement, the examiner trained participants about the protocol (movements, directions, repetitions, and sequence) and required stabilizations [18], and patients carried out five full movements to get used to them and to warm up tissues. If the examiner observed any compensatory movement due to a bad stabilization, participants were asked to do an additional attempt without compensation.

The Veloflex-IDA’s measurements began by placing the markers on representative anatomical landmarks, asking the participants to keep the final position of the movement being assessed. The markers were placed on three different points: the joint axis and two references, or as an alternatively to the latter, between a bone segment and the horizontal/vertical imaginary line drawn on the joint axis. Then, from the starting position, the participants were asked to reach the end of the range, maintain it for 3 s and go back to the neutral position.

Each movement was measured three times, with 5 s rest between them. Next, the same procedure was performed in the opposite direction. A rest of 60 s was given between movements in different planes or joints. In addition, in the first session, consecutive measurements were made with Veloflex-IDA and the goniometer while subjects maintained the position for 3 s at the end of the ROM. The order of measurement between the two tools was randomized, placing the Veloflex-IDA’s markers before or after the goniometer’s measurements according to the assigned order.

### 2.3. Sample Size Estimation

Sample size was calculated using the formula for reliability studies based on confidence intervals (CIs) described by [19]. With the number of instruments (k) equal to 2, the CI around r (the reliability coefficient) of 0.05, and an estimated r of 0.95, the sample size (n) was calculated to be 25 participants.

### 2.4. Statistical Analysis

Participant characteristics and ROM values (degrees) are presented as mean (SD) or percentages, as appropriate. The mean between repetitions was used for analyses. SPSS (version 24; SPSS Inc., Chicago, IL, USA) was used to perform all statistical analyses. First, to analyze the concurrent validity and the agreement between instruments, Pearson’s product moment correlation coefficient (r) with 95% confidence intervals (CI) and Bland–Altmann plots were used, respectively. Furthermore, the following values were calculated: the upper and lower limits of agreement (LoA), the mean and SD of the difference between instruments (in both with absolute difference and percentages) regarding the goniometer values. Those two last statistics are respectively called bias and imprecision. Second, to analyze the Veloflex-IDA reliability, the values examined were (1) the relative reliability using the intra-class correlation (ICC) and (2) the absolute reliability by calculating the standard error of measurement (SEM) and the minimal detectable change (MDC). The reliability was classified as excellent (ICC > 0.90), good (ICC = 0.76–0.90), moderate (ICC = 0.51–0.75), and poor (ICC < 0.50) [20]. MDC was calculated for the 95% CI as MDC_95_ = SEM × 1.96 × $\sqrt{2}$, where SEM = $\mathrm{SD}\sqrt{\left(1-\mathrm{ICC}\right)}$.

## 3. Results

### 3.1. Participants

Asymptomatic participants showed an average age of 34.8 years (SD = 12.5) with a BMI of 25.3 kg/m^2^ (SD = 5.9). Symptomatic participants showed a mean age of 44.7 years (SD = 17.5) with a BMI of 24.7 kg/m^2^ (SD = 1.5). Fifteen subjects presented shoulder disorder and 10 subjects showed wrist disorder, with an average pain of 5.1 out of 10. The average duration of symptoms in this group was 6.2 weeks (Table 1).

### 3.2. Concurrent Validity and Agreement

**Figure 2:**
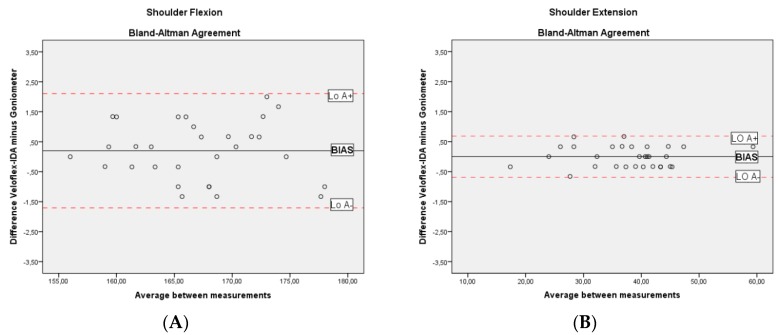

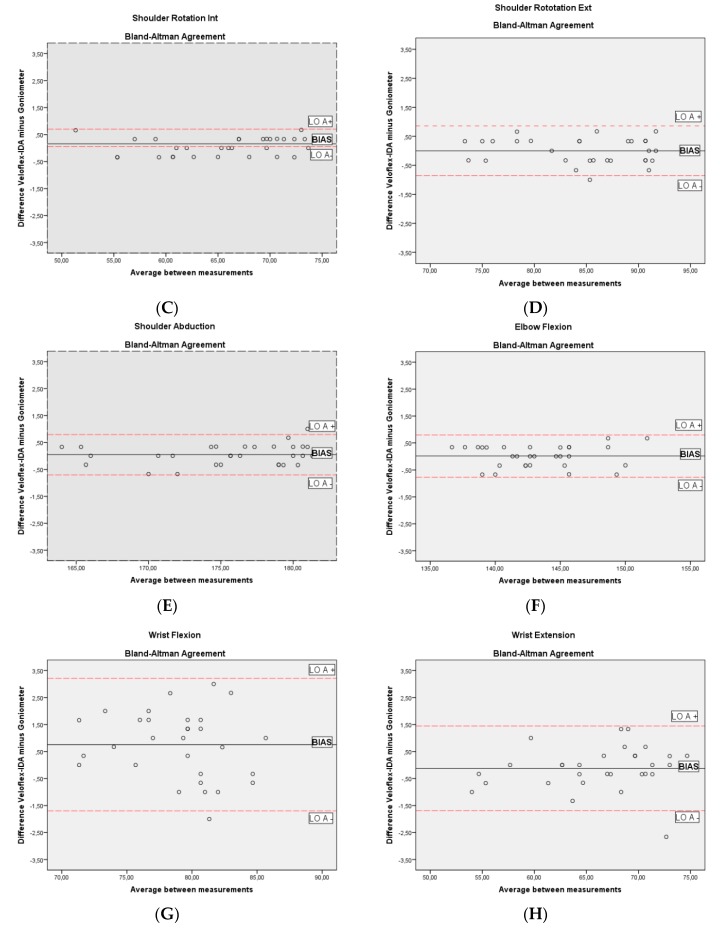

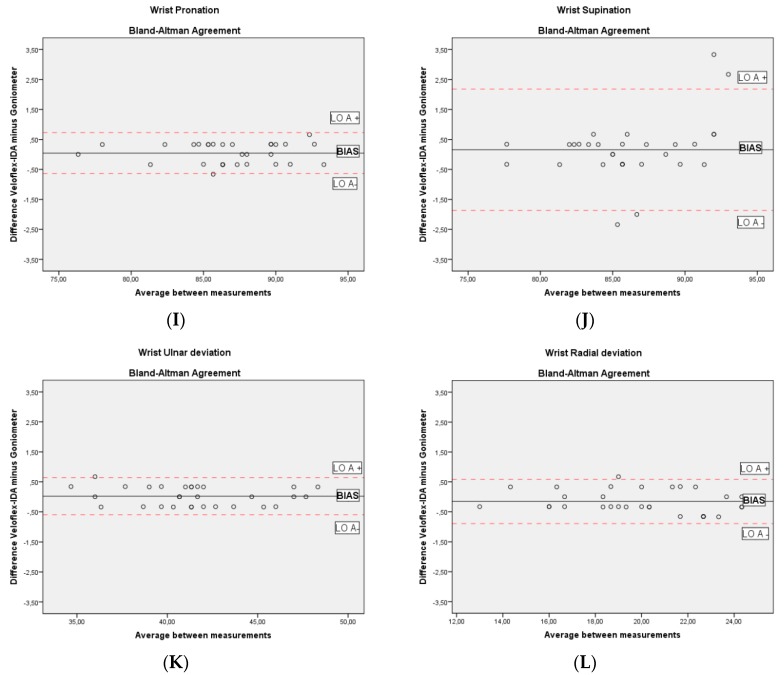
Bland–Altman plots for Veloflex infrared dynamic angle-meter (Veloflex-IDA) and goniometer during (**A**) shoulder flexion; (**B**) shoulder extension; (**C**) shoulder internal rotation; (**D**) shoulder external rotation; (**E**) shoulder abduction; (**F**) elbow flexion; (**G**) wrist flexion; (**H**) wrist extension; (**I**) wrist pronation; (**J**) wrist supination; (**K**) wrist ulnar deviation; (**L**) wrist radial deviation.

Both instruments showed an excellent correlation for movements of the shoulder (*r* range = 0.986 to 0.999), elbow (*r* = 0.995), and wrist (*r* range = 0.990 to 0.996) (Table 1). Bland–Altman plots are displayed in Figure 2. Both instruments provided an almost perfect agreement with small mean ‘bias’ (<1%) and ‘imprecision’ (<1.9%) compared to the goniometer.

### 3.3. Reliability

Table 2, Table 3 and Table 4 show intra- and inter-rater reliability analysis data for the Veloflex-IDA in asymptomatic and symptomatic participants. Relative reliability analysis showed excellent reliability both for intra-rater reliability (ICC range = 0.95 to 0.99) and for inter-rater reliability (ICC range = 0.90 to 0.98) in both groups. Absolute intra-rater reliability analysis showed SEMs < 3.17% and MCD < 2.41° in asymptomatic participants and SEMs < 5.19% and MCDs < 16.48° in symptomatic for all movements. For inter-rater reliability, SEMs < 3.05% and MCDs < 3.04° in asymptomatic and SEMs < 6.30% and MCDs < 16.68° in symptomatic were found.

## 4. Discussion and Conclusions

The present study showed that Veloflex-IDA is a valid and reliable tool for the ROM assessment of upper limb joints’ ROM. In addition, excellent inter-rater reliability values were obtained after basic training in its use.

Veloflex-IDA and goniometer showed a very high correlation between them, with small mean bias and imprecision. In addition, this was observed in all movements performed, showing a high consistency between instruments independently of the test. Our findings are slightly superior to those of other studies that have examined the validity of other instruments with the goniometer as a gold standard [12,21]. In the same way, compared to shoulder and wrist studies where the goniometer was used as a reference, our LoAs results were lower [10,12,14,22]. This could be due to their use of a mobile phone application to measure the ROM. Accordingly, that system implies to attach the mobile phone in the body segment doing the movement, while the Veloflex-IDA does not need more than the placement of the markers.

The intra-rater reliability obtained in the ROM measurements proved to be excellent, with minimal differences between movements in both the ICCs and in the SEM variations. In relation to the relative reliability statistics, our ICCs are similar [12,15] or superior [8,13,21] to shoulder studies and slightly superior to others performed on the wrist [14]. Furthermore, absolute reliability statistics proved to be superior to those studies that used a mobile phone or electrogoniometer as a measurement tool both in healthy subjects [8,13,21] and in subjects with wrist limitations [14]. Only SEMs and MCDs are similar to the use of video processing tools (e.g., Kinovea) for ROM shoulder measurements [15]. To our knowledge, our study was the first to compare the tool’s reliability to assess the ROM of shoulder, elbow, and wrist joints. Reliability comparisons among joints showed ICCs and SEM% slightly higher for shoulder movements than for the wrist. These differences can be explained by the differences between their ROM values, since previous studies found similar results with better reliability for those movements with higher ROMs [23,24].

The inter-rater reliability behaved in a similar way to the intra-rater, with higher values in our study for both relative and absolute reliability statistics compared to those studies that used mobile phones or electrogoniometers [8,10,13,14,21] and similar with video processing tools [15]. Consequently, we believe that those instruments that need to be placed on the body segment to measure the ROM decrease their reliability, because of the possible error in their placement and the need for the instrument to remain stable during the movement. Inter-rater values were slightly worse than intra-rater reliability for all tests. This finding agrees on other shoulder [12,15] and wrist [14] studies. We believe that this is not due to the difference between the examiners’ experience, since given a basic training to the newly graduated examiner was enough to obtain high reliability values as in previous studies [21].

This study provides useful information on Veloflex-IDA validity and reliability to measure the ROM of the upper limb joints, despite being subject to limitations. The main limitation was that the participants were predominantly young and healthy, limiting the generalization of the measurements to other populations. Thereby, future studies should study the methodology used to measure populations with disorders. In addition, only the upper limb joints’ ROM was examined. Thus, future studies should evaluate the Veloflex-IDA’s reliability for other joints.

In conclusion, Veloflex-IDA is a valid and reliable alternative to measure the ROM of the upper limb joints and it can be used in clinical practice and research after basic training.

## Figures and Tables

**Table 1:** Characteristics of the participants.

	Asymptomatic (*n* = 30)	Symptomatic (*n* = 20)
Age (years)	35.3 (12.8)	44.7 (17.5)
BMI (kg/m^2^)	25.7 (6.2)	24.7 (1.5)
Gender	Males (*n* = 18)	Males (*n* = 13)
Pain (0–10/10)	0	5.1 (1.9)
Duration of symptoms (weeks)	-	6.2 (3.6)
Shoulder disorder	-	*n* = 15
Elbow disorder	-	*n* = 0
Wrist disorder	-	*n* = 10

**Table 2:** Validity between Veloflex-IDA and goniometer to measure the range of motion of the upper limb joints.

Movement	Goniometer (SD)	Veloflex-IDA (SD)	Pearson Coefficient	LoA − (%)	LoA + (%)	Mean Difference (%)	SD Difference (%)
ShF	166.87° (5.80°)	167.07° (5.67°)	0.986	−1.70° (1.02%)	2.11° (1.26%)	0.20° (0.10%)	0.97° (0.60%)
ShE	37.78° (8.28°)	37.78° (8.29°)	0.999	−0.69° (1.81%)	0.68° (1.81%)	−0.01° (0.01%)	0.35° (1.10%)
ShIR	65.44° (6.07°)	65.49° (6.14°)	0.999	−0.59° (0.89%)	0.70° (1.06%)	0.05° (0.10%)	0.32° (0.50%)
ShER	84.71° (6.085)	84.71° (6.01°)	0.997	−0.86° (1.01%)	0.86° (1.01%)	−0.01° (0.01%)	0.44° (0.5%)
hAB	175.50° (5.15°)	175.54° (5.22°)	0.997	−0.70° (0.39%)	0.79° (0.44%)	0.04° (0.01%)	0.38° (0.20%)
ElbF	143.35° (3.84°)	143.36° (3.84°)	0.995	−0.77° (0.53%)	0.79° (0.55%)	0.01° (0.01%)	0.39° (0.30%)
WrF	78.18° (4.30°)	78.93° (3.89°)	0.958	−1.70° (2.17%)	3.21° (4.10%)	0.75° (1%)	1.25° (1.6%)
WrE	66.37° (5.65°)	66.24° (5.71°)	0.99	−1.69° (2.54%)	1.44° (2.17%)	−0.12° (0.2%)	0.80° (1.2%)
WrPr	86.83° (3.93°)	86.87° (3.93°)	0.996	−0.64° (0.73%)	0.72° (0.83%)	0.04° (0.10%)	0.34° (0.40%)
WrSup	85.93° (3.77°)	86.08° (4.01°)	0.967	−1.87° (2.17%)	2.18° (2.53%)	0.15° (0.20%)	1.03° (1.20%)
WrUlDev	41.49° (3.63°)	41.51° (3.57°)	0.996	−0.60° (1.43%)	0.64° (1.54%)	0.02° (0.1%)	0.31° (0.8%)
WrRadDev	20.02° (3.08°)	19.87° (3.08°)	0.993	−0.90° (4.47%)	0.58° (2.92%)	−0.15° (−0.7%)	0.38° (1.9%)

SD: standard deviation; LoA: limit of agreement; ShF: shoulder flexion; ShE: shoulder extension; ShIR: shoulder internal rotation; ShER: shoulder external rotation; ShAB: shoulder abduction; ElbF: elbow flexion; WrF: wrist flexion; WrE: wrist extension; WrPr: wrist pronation; WrSup: wrist supination; WrUlDev: wrist ulnar deviation; WrRadDev: wrist radial deviation.

**Table 3:** Reliability of Veloflex-IDA to measure the range of motion of the upper limb joints in symptomatic participants.

	Intra-Rater				Inter-Rater			
Movement	Retest (SD)	ICC (95% CI)	SEM (SEM%)	MDC	Test (SD)	ICC (95% CI)	SEM (SEM%)	MDC
ShF	166.87° (5.80°)	0.99 (0.98 to 0.99)	5.32° (4.65%)	1.31°	166.62° (6.54°)	0.96 (0.93 to 0.98)	1.09° (0.66%)	3.04°
ShE	37.69° (8.39°)	0.99 (0.99 to 0.99)	0.98° (2.87%)	1.44°	37.69° (7.98°)	0.98 (0.97 to 0.99)	0.85° (2.26%)	2.36°
ShIR	65.53° (5.86°)	0.99 (0.98 to 0.99)	2.08° (4.02%)	1.38°	65.46° (5.95°)	0.98 (0.97 to 0.99)	0.66° (1.01%)	1.85°
ShER	84.60° (5.86°)	0.99 (0.98 to 0.99)	2.81° (4.24%)	1.46°	83.73° (6.07°)	0.98 (0.97 to 0.99)	0.65° (0.77%)	2.74°
ShAB	175.08° (5.19°)	0.98 (0.97 to 0.99)	5.95° (4.93%)	1.50°	174.41° (5.06°)	0.96 (0.93 to 0.98)	0.91° (0.52%)	2.75°
ElbF	142.62° (3.92°)	0.95 (0.91 to 0.98)		2.22°	142.65° (3.88°)	0.92 (0.85 to 0.96)	1.04° (0.73%)	2.53°
WrF	78.18° (4.30°)	0.97 (0.95 to 0.99)	2.14° (3.08%)	1.75°	78.39° (3.91°)	0.95 (0.90 to 0.98)	0.85° (1.07%)	2.35°
WrE	65.82° (6.17°)	0.98 (0.96 to 0.99)	2.47° (5.19%)	2.25°	66.09° (6.09°)	0.98 (0.96 to 0.99)	0.76° (1.15%)	2.11°
WrPr	85.01° (3.95°)	0.96 (0.92 to 0.98)	1.14° (1.40%)	2.17°	85.77° (3.99°)	0.90 (0.81 to 0.96)	1.19° (1.39%)	2.52°
WrSup	85.01° (3.77°)	0.95 (0.89 to 0.97)	0.88° (1.03%)	2.41°	85.40° (3.43°)	0.94 (0.88 to 0.97)	0.85° (1.03%)	2.61°
WrUlDev	40.71° (3.62°)	0.96 (0.92 to 0.98)	1.01° (2.98%)	1.91°	41.34° (4.22°)	0.96 (0.92 to 0.98)	0.76° (1.84%)	2.12°
WrRadDev	19.62° (2.95°)	0.96 (0.91 to 0.98)	0.84° (4.82%)	1.74°	20.18° (2.94°)	0.96 (0.91 to 0.98)	0.61° (3.05%)	1.69°

SD: standard deviation; ICC: intraclass correlation coefficient; CI: confidence interval; SEM: standard error of measurement; MDC: minimum detectable change; ShF: shoulder flexion; ShE: shoulder extension; ShIR: shoulder internal rotation; ShER: shoulder external rotation; ShAB: shoulder abduction; ElbF: elbow flexion; WrF: wrist flexion; WrE: wrist extension; WrPr: wrist pronation; WrSup: wrist supination; WrUlDev: wrist ulnar deviation; WrRadDev: wrist radial deviation.

**Table 4:** Reliability of Veloflex-IDA to measure the range of motion of the upper limb joints in symptomatic participants.

	Intra-Rater				Inter-Rater			
Shoulder Disorder (*n* = 15)	Test (SD)/Retest (SD)	ICC (95% CI)	SEM (SEM%)	MDC	Test (SD)	ICC (95% CI)	SEM (SEM%)	MDC
ShF	114.49° (53.28°)/114.65° (54.00°)	0.99 (0.98 to 0.99)	5.32° (4.65%)	14.77°	114.76° (53.68°)	0.99 (0.99 to 0.99)	5.37° (4.68%)	14.88°
ShE	34.08° (9.80°)/33.93° (9.82°)	0.99 (0.97 to 0.99)	0.98° (2.87%)	2.71°	34.08° (9.50°)	0.99 (0.97 to 0.99)	0.95° (2.79%)	2.63°
ShIR	51.74° (20.78°)/51.87° (21.64°)	0.99 (0.98 to 0.99)	2.08° (4.02%)	5.76°	51.98° (21.27°)	0.99 (0.98 to 0.99)	2.13° (4.09%)	5.89°
ShER	66.21° (28.10°)/66.21° (28.1°5)	0.99 (0.99 to 0.99)	2.81° (4.24%)	7.79°	66.36° (27.87°)	0.99 (0.99 to 0.99)	2.73° (4.11%)	7.56°
ShAB	120.49° (59.46°)/120.63° (60.35°)	0.99 (0.99 to 0.99)	5.95° (4.93%)	16.48°	120.49° (60.17°)	0.99 (0.99 to 0.99)	6.02° (4.99%)	16.68°
**Wrist disorder (*n* = 10)**								
WrF	69.40° (21.36°)/68.66° (21.09°)	0.99 (0.98 to 0.99)	2.14° (3.08%)	5.92°	68.66° (21.08°)	0.99 (0.98 to 0.99)	2.11° (3.07%)	5.84°
WrE	47.52 (24.58°)/46.64° (24.19°)	0.99 (0.99 to 0.99)	2.47° (5.19%)	6.84°	46.88° (24.48°)	0.99 (0.99 to 0.99)	2.45° (5.22%)	6.79°
WrPr	80.98 (11.38°)/80.06° (10.81°)	0.99 (0.98 to 0.99)	1.14° (1.40%)	3.15°	80.33° (11.27°)	0.99 (0.97 to 0.99)	1.13° (1.40%)	3.12°
WrSup	84.87 (5.09°)/83.88° (5.13°)	0.97 (0.89 to 0.99)	0.88° (1.03%)	2.44°	83.85° (4.93°)	0.95 (0.85 to 0.99)	1.10° (1.31%)	3.05°
WrUlDev	33.70 (10.04°)/35.82° (10.79°)	0.99 (0.97 to 0.98)	1.01° (2.98%)	2.78°	35.70° (10.52°)	0.99 (0.97 to 0.99)	1.05° (2.95%)	2.91°
WrRadDev	17.37 (5.92°)/16.64° (5.57°)	0.98 (0.92 to 0.99)	0.84° (4.82%)	2.32°	16.73° (5.27°)	0.96 (0.85 to 0.99)	1.05° (6.30%)	2.92°

SD: standard deviation; ICC: intraclass correlation coefficient; CI: confidence interval; SEM: standard error of measurement; MDC: minimum detectable change; ShF: shoulder flexion; ShE: shoulder extension; ShIR: shoulder internal rotation; ShER: shoulder external rotation; ShAB: shoulder abduction; ElbF: elbow flexion; WrF: wrist flexion; WrE: wrist extension; WrPr: wrist pronation; WrSup: wrist supination; WrUlDev: wrist ulnar deviation; WrRadDev: wrist radial deviation.
